# *Medicago truncatula PHO2* genes have distinct roles in phosphorus homeostasis and symbiotic nitrogen fixation

**DOI:** 10.3389/fpls.2023.1211107

**Published:** 2023-06-13

**Authors:** Raul Huertas, Ivone Torres-Jerez, Shaun J. Curtin, Wolf Scheible, Michael Udvardi

**Affiliations:** ^1^ Noble Research Institute LLC, Ardmore, OK, United States; ^2^ United States Department of Agriculture, Plant Science Research Unit, St. Paul, MN, United States; ^3^ Department of Agronomy and Plant Genetics, University of Minnesota, St. Paul, MN, United States; ^4^ Center for Plant Precision Genomics, University of Minnesota, St. Paul, MN, United States; ^5^ Center for Genome Engineering, University of Minnesota, St. Paul, MN, United States

**Keywords:** phosphorus, symbiotic nitrogen fixation (SNF), *Medicago truncatula*, *PHO2*, nitrogen

## Abstract

Three *PHO2*-like genes encoding putative ubiquitin-conjugating E2 enzymes of *Medicago truncatula* were characterized for potential roles in phosphorous (P) homeostasis and symbiotic nitrogen fixation (SNF). All three genes, *MtPHO2A, B and C*, contain miR399-binding sites characteristic of *PHO2* genes in other plant species. Distinct spatiotemporal expression patterns and responsiveness of gene expression to P- and N-deprivation in roots and shoots indicated potential roles, especially for *MtPHO2B*, in P and N homeostasis. Phenotypic analysis of *pho2* mutants revealed that MtPHO2B is integral to Pi homeostasis, affecting Pi allocation during plant growth under nutrient-replete conditions, while MtPHO2C had a limited role in controlling Pi homeostasis. Genetic analysis also revealed a connection between Pi allocation, plant growth and SNF performance. Under N-limited, SNF conditions, Pi allocation to different organs was dependent on MtPHO2B and, to a lesser extent, MtPHO2C and MtPHO2A. MtPHO2A also affected Pi homeostasis associated with nodule formation. Thus, *MtPHO2* genes play roles in systemic and localized, i.e., nodule, P homeostasis affecting SNF.

## Introduction

Nitrogen (N) and phosphorus (P) are essential macronutrients for plant growth and development. Low availability of these nutrients in most soils limits crop production necessitating the use of fertilizers to secure food production. Symbiotic nitrogen fixation (SNF) in legumes is the primary natural source of nitrogen in agroecosystems, although industrial nitrogen-fertilizers now provide most of the nitrogen for crop production. Use and loss to the environment of industrial N-fertilizer is not sustainable and more needs to be done to boost the use of legumes and N derived from SNF in agriculture to remedy this (Udvardi et al., 2021).

SNF results from a mutualistic symbiosis between soil bacteria, called rhizobia, and legumes during which the bacteria reduce atmospheric di-nitrogen into ammonia within specialized root organs called nodules. In exchange for ammonia provided to the plant, the bacteria receive carbon (C) in the form of organic acids and other nutrients, including P for instance (Udvardi & Poole, 2013). Complex regulatory networks have evolved to control acquisition and allocation of C, N, P and other essential nutrients for optimal growth, development and functioning of plant organs and the plant as a whole (Gautrat et al., 2021; Helliwell, 2022), although our understanding of these networks remains incomplete.

SNF is sensitive to environmental stress, including P-deficiency. Nodules contain relatively high concentrations of P especially in nucleic acids (plant and bacterial DNA and RNA), which underpin protein synthesis and high metabolic activity, including SNF (Sulieman et al., 2013; Cabeza et al., 2014). P limitation severely inhibits root nodule organogenesis and SNF (Hernandez et al., 2009). Maintenance of P homeostasis in nodules is considered a main adaptive strategy to maintain symbiotic performance under P-deficiency, although underlying mechanisms are poorly understood. (Cabeza et al., 2014; Nasr Esfahani et al., 2017).

In non-legumes, there is growing evidence for crosstalk between P and N regulation of nutrient acquisition, growth and metabolism (Hu et al., 2019; Medici et al., 2019; Ueda et al., 2020). In Arabidopsis and other species, P homeostasis is systemically regulated by the transcriptional activators Phosphate Starvation Response 1 (PHR1/PHL) (Rubio et al., 2001; Bustos et al., 2010), the negative regulator SPX-like (Puga et al., 2014; Wang et al., 2014), and tuned by the balance of specific microRNAs (miR399 and miR827) (Chiou et al., 2006; Lin et al., 2013) and long non-coding RNAs (IPS-like) (Franco-Zorrilla et al., 2007) in coordination with PHOSPHATE2 (PHO2) (Aung et al., 2006; Bari et al., 2006; Lin et al., 2008). PHO2 is a ubiquitin-conjugating (UBC) E2 enzyme involved in the degradation of multiple type of Pi transporters including members of the PHT1/PT (PHOSPHATE TRANSPORTER 1) protein family, PHOSPHATE 1 (PHO1) and PHF1 (PHOSPHATE TRANSPORTER TRAFFIC FACILITATOR 1) (Bari et al., 2006; Liu et al., 2012; Cao et al., 2014; Park et al., 2014; Ouyang et al., 2016; Pacak et al., 2016). The PHO2-miR399-IPS1 and PHO2-NLA-miR827 regulatory modules (Franco-Zorrilla et al., 2007; Kant et al., 2011; Lin et al., 2013) function independently but cooperatively, regulating acquisition and root-to-shoot translocation of Pi in response to P and N availability, protecting aboveground organs from excessive Pi accumulation. The physiological role of PHO2 protein in maintaining whole-plant Pi-homeostasis has been described for rice (Cao et al., 2014), wheat (Ouyang et al., 2016) and Arabidopsis (Bari et al., 2006). Further, Arabidopsis PHO2 is considered a local and systemic integrator of N availability in phosphate systemic signaling (Medici et al., 2019).

Although the mechanisms of N and P crosstalk in legumes related to nodule development and SNF are largely unknown, there is evidence that systemic signaling pathways controlling N fixation and acquisition are linked to phosphate systemic signaling. Phosphorus deficiency influence rhizobial infection and nodulation through miR2111/Too Much Love (TML), PHR (Phosphate Starvation Response) - RICs (Rhizobium-induced CLE Peptides) - NARK (Nodulation Autoregulation Receptor Kinase) and through PHR depending on P homeostasis regulatory modules in legumes (Gautrat et al., 2021; Zhong et al., 2023 and references therein). Some Phosphate Transporter (PHT/PT) and PHOSPHATE1 (PHO1)-type P transporters, downstream targets of PHR transcription factors, have been assigned an important role in maintaining Pi homeostasis in nodules, supporting SNF (Qin et al., 2012; Chen et al., 2019; Nguyen et al., 2021). Likewise, two alfalfa PHO2 genes have been implicated in systemic P-homeostasis, although their roles during symbiosis have not been explored (Miller et al., 2022) A *Medicago truncatula PHO2*-like gene contributes to quantitative variation in nodulation in this species, but the underlying mechanism remains unknown (Curtin et al., 2017). *Medicago truncatula* has two other *PHO2*-like genes, although their roles, if any, in P-homeostasis and SNF also remain unknown (Miller et al., 2022).

Here, we explore the roles of the three *Medicago truncatula PHO2-like* genes in P homeostasis and SNF. Our results implicate PHO2 proteins in systemic Pi homeostasis and the support of SNF.

## Materials and methods

### Plant material

*Medicago truncatula* ecotype R108 (HM340) was used in all experiments as a wild-type control as this is the genetic background of the *Tnt1*, CRISPR/Cas9 and TALEN *pho2* mutants. Offspring of the CRISPR/Cas9 line WPT210-9, described previously (Curtin et al. (2017), were screened to isolate homozygous *pho2-A*_CRISPR_ and *pho2-B*_CRISPR_ mutants. Similarly, offspring of TALEN line WPT52-4-8 (Cermak et al. (2017), were used to identify the homozygous mutant *pho2-B*_TALEN_. Offspring of self-pollinated plants from the WPT210-9 and WPT52-4-8 lines were genotyped by combining PCR amplification and NlaIV and HaeIII restriction enzyme digestion assays, respectively. Changes in genomic DNA were confirmed by Sanger sequencing of undigested PCR products of the homozygous mutant lines and wild-type plants. Sequence comparisons were performed using Geneious software.

The *pho2-A*_Tnt1_ homozygous line was obtained from *Tnt1* line NF12360 (Curtin et al., 2017). *Tnt1* line NF16248 was used to isolate the homozygous exonic mutant allele *pho2-C*_Tnt1_ by PCR genotyping. *Tnt1*-specific and gene-specific primers are listed in **Supplementary Table 2**. *Pho2-A*_CRISPR_, *pho2-B*_CRISPR_ and *pho2-C*_Tnt1_ were the mutant alleles selected for phenotypic analysis, while the *pho2-A*_Tnt1_ and *pho2-B*_TALEN_ mutant alleles were used to confirm phenotypes **(** **Supplementary Figure 6** **)**.

### Plant growth under non-symbiotic and symbiotic conditions

Seeds were sterilized, scarified, and stratified as described before (Kryvoruchko et al., 2018). Seedlings with fully opened cotyledons and similar radicles were individually transferred into 2” x 7” plastic cones (Stuewe & Sons Inc.) containing a mixture (3:1, v/v) of sterilized Turface (calcined [illite] clay) and Vermiculite (Sun Gro Horticulture). Seedlings were fertilized with one-half-strength B&D solution (Broughton & Dilworth, 1971). Seven days after transplanting, seedlings were watered with full-strength modified B&D nutrient solution. Nitrogen was supplied as a 2:1 mixture of KNO_3_ and NH_4_NO_3_, while phosphorus was supplied as KH_2_PO_4_. The different treatments and final concentrations were “control” (8 mM N and 0.5 mM P), “reduced-P” (8 mM N and 20 μM P), “control_sym_” (0.25 mM N and 0.5 mM P) and “reduced-P_sym_” (0.25 mM N and 20 μM P). K_2_SO_4_ was used to balance the potassium concentration in the reduced-P and reduced-P_sym_ solutions. All other macro- and micro-nutrients of the B&D nutrient solution were provided as specified (Broughton & Dilworth, 1971).

For the non-symbiotic experiments, plants were watered with control or reduced-P nutrient solutions, while for the symbiotic experiments they were inoculated with 50 mL suspension (OD_600_ ∼0.02) of *Sinorhizobium meliloti* strain 1021 (Meade et al., 1982) in control_sym_ or reduced-P_sym_ nutrient solutions. Plants were grown under controlled conditions of light (200 μmol m^-2^ s^-1^, 16h day/8h night), constant temperature of 22°C, and 40% relative humidity, and irrigated twice per week - once with the corresponding nutrient solution and once with B&D without N or P, to avoid accumulation of N or P. Plants were harvested four weeks after starting treatments. Each plant was removed from its cone and the root carefully washed with water to remove substrate while avoiding loss of roots and nodules. Independent plant tissues were frozen in liquid nitrogen and stored at −80°C for later use, or oven dried at 60°C and weighed. Total plant dry weight (DW) was the sum of the shoot and root dry weights.

### Symbiotic nitrogen fixation traits

The acetylene reduction assay (ARA) was carried out as previously described (Hardy et al., 1968). Briefly, four weeks after inoculation, entire root systems were transferred onto sterile Whatman paper strips placed inside 12-mL glass vials containing 2 mL of sterile distilled water. The tubes were sealed with rubber stoppers. Each tube contained roots from independent plants. Samples were incubated in dark in the presence of 10% (v/v) acetylene at 28°C for up to 16 h. Ethylene and acetylene concentrations were measured using an Agilent 7890A gas chromatograph (Agilent Technologies). Serial dilutions of a known quantity of ethylene were used to make standard curves of GC chromatogram peak area to calculate the amount of ethylene produced. The amount of ethylene produced was determined by measuring the area of the ethylene peak relative to background. Nitrogenase activity was calculated as the amount of ethylene produced per unit root dry weight. The number and biomass of the nodules was determined by detaching them from the roots.

### RNA isolation and quantitative PCR (qPCR) analyses

Four weeks after treatments, roots, shoots, and nodules were collected into liquid nitrogen. After grinding in liquid nitrogen, total RNA was extracted using the TRIzol reagent (Life Technologies). Residual genomic DNA was removed using Turbo DNase I (Ambion). RNA was quantified using a Nanodrop Spectrophotometer ND-100 (NanoDrop Technologies). For qPCR, reverse transcription was carried out using SuperScript III Reverse Transcriptase (Invitrogen) and oligo(dT)20 primer, as describe previously. Transcript levels were normalized using the geometric mean of three housekeeping genes, MtPI4K (Medtr3g091400), MtPTB2 (Medtr3g090960), and MtUBC28 (Medtr7g116940), whose transcript levels were stable across all the samples analyzed (Kakar et al., 2008). Three biological replicates were included per gene. qPCR cycle threshold (Ct) values were analyzed using the ΔΔCt method (Livak & Schmittgen, 2001). Primer sequences used in this analysis are listed in **Supplementary Table 2**. Sequence alignments and the design of gene specific primers were performed using Geneious software.

### Bioinformatics and phylogenetic analysis

The genome assembly of Jemalong A17 (Mt4.0 v1) and Medicago R108 (v0.95) ecotypes from Phytozome and the Legume Information System (LIS) were consulted to retrieve DNA sequences and gene structures. The Integrative Genomics Viewer (IGV) software (https://igv.org) was used to visualize the original raw RNA-seq data used for the MtSSPdb (https://mtsspdb.zhaolab.org/database) (Boschiero et al., 2020) and confirm the 5’ and 3’ UTR regions as well as the expression profiles.

Precursor sequences of the pre-miR399s from several plant species were obtained from miRBase (www.mirbase.org) and used to identify *Medicago truncatula* miR399. The miR399 sequences and the potential miR399-binding sites (miR399BS) were validated using psRNATarget with default parameters (Dai et al., 2018). The consensus miR399 sequence, miR399 sequence logo, potential PHO-like ([G(G/T/A) (C/T/A)GTGG]; Mukatira et al., 2001) and P1BS (GnATATnC; Rubio et al., 2001) cis-regulatory elements were generated using Geneious software.

Protein sequences were extracted from Phytozome and NCBI Protein databases. The circular phylogenetic tree was constructed from a ClustalW multiple sequence alignment of the full‐length protein sequences in Geneious software using Juker-Cantor as the genetic distance model and the unweighted pair group method with arithmetic mean (UPGMA) as a tree build method with 500 replicates and 60% of support threshold. The gene IDs encoding each protein are described in **Supplementary Table 1**.

### Measurements of soluble phosphate concentration

Soluble inorganic phosphate (Pi) was measured in four-week-old plants after treatments according to Miller et al. (2022), with minor modifications. Briefly, frozen tissue samples (leaves, roots and nodules) were ground. Deionized water was added to the homogenized samples, mixed, and centrifuged at 13,000 g for 3 minutes, and the clarified supernatant was transferred to a clean tube to quantify Pi content. Aliquots were diluted appropriately and mixed in 96 deep-well plates with HCl and malachite green reagent. After 15 minutes of incubation at room temperature, light absorbance was measured at 660 nm. The sample Pi concentration was determined by reference to a calibration curve using K_2_HPO_4_. Pi concentration was calculated based on fresh weight of samples. Measurements were performed in triplicate in three independent biological replicates.

### Statistical analysis and graphs

Data on gene expression, biomass, Pi content, etc. were analyzed statistically for mean comparisons, between wild type and mutant alleles or between control and treatment conditions, by one-way analysis of variance (ANOVA) and *t*-tests (P<0.05). Statistical analyses and graphs were generated using GraphPad Prism software.

## Results

### Identification of *PHO2* genes in *Medicago truncatula* and phylogenetic analysis

**Figure 1:**
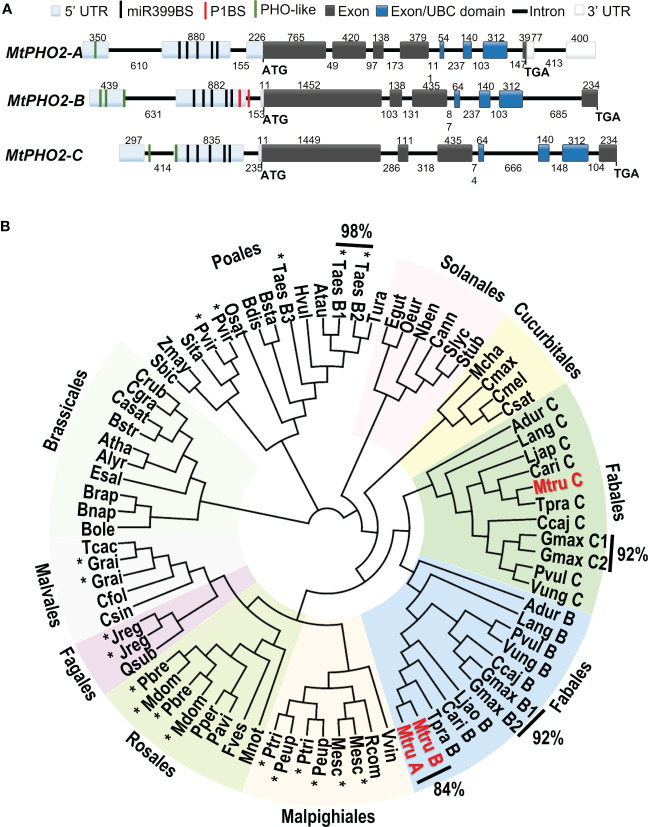
Gene structure and phylogenetic relationships of *Medicago truncatula PHO2*-like genes. **(A)** Gene structure of the three *PHO2* genes present in the A17 *Medicago truncatula* genome (Mt4.0v1) and validated using RNA-seq data. Only splicing variant *MtPHO2-A.2* is shown here (see **Supplementary Figure 1A** for other slicing variants). Exons are shown as grey or darker blue boxes, with the latter encoding the ubiquitin-conjugating (UBC) domain (see also **Supplementary Figure 1B**). UTRs are shaded light blue (5’ of the coding sequence) or white boxes (3’ of the coding sequence). The black, green and red lines depict the position within the 5’ UTR of the five potential miR399-binding sites (miR399BS), PHO-like elements, and the PHR1 binding sites (P1BS). Promoters and cis-regulatory motifs are detailed in **Supplementary Figure 2**. Gene structures are drawn to scale, and the associated numbers indicate sizes (numbers above exons and below introns). **(B)** Phylogenetic tree of the *PHO2-like* gene family in plants. The different orders are marked next to different colored backgrounds. The *Medicago truncatula PHO2* genes are indicated in red (MtruA, *MtPHO2-A*; MtruB, *MtPHO2-B*; MtruC, *MtPHO2-C*). The * indicates gene duplication events outside of the order Fabales. Values outside of the Phylogenetic tree show the percentages of homology between MtPHO2A and B proteins as well as some other plant species. The protein sequences were extracted from Phytozome and NCBI Protein databases. The circular phylogenetic tree was constructed from a ClustalW alignment of the full‐length protein sequences in Geneious software using Juker-Cantor as the genetic distance model and UPGMA as a tree build method with 500 replicates and 60% of support threshold. The gene IDs encoding each protein are described in **Supplementary Table S1**.

*M. truncatula PHO2* genes were identified *via* BLASTP searches using the Phytozome and the Legume Information System (LIS) as databases and known plant PHO2 proteins as queries **(** **Supplementary Table 1** **)**. Three separate genes were identified and named *MtPHO2-A* (Medtr4g020620), *MtPHO2-B* (Medtr2g013650) and *MtPHO2-C* (Medtr4g088835) **(** **Figure 1A** **)**, keeping Medtr4g020620 as -A, the first *PHO2* gene described in *M. truncatula* (Curtin et al., 2017). Note that Wang et al. (2017) described *Medtr2g013650* as the only *PHO2* gene in Medicago, which we named *MtPHO2-B* to distinguish it from the other two *PHO2* genes we identified.

Detailed *in silico* analysis of the annotated DNA sequences and visualization of raw RNA-seq data (Boschiero et al., 2020), with the Integrative Genomics Viewer (IGV) (Robinson et al., 2011), were used to validate gene structures. These three *MtPHO2* genes shared the typical number (7-9) and arrangement of exons and regulatory elements in the proximal promoter region/5’-UTR **(** **Figure 1A** **)**.

Analyses of the 5′-UTR of the three *MtPHO2* genes showed five putative miR399-binding sites (miR399-BS) and PHO-like elements, while only t*MtPHO2-B* presented putative PHR1 binding sites (P1BS) in its 5′-UTR **(** **Figure 1A**; **Supplementary Figure 2A** **)**. Using mature and stem-loop sequences of known miRNA399s obtained from miRbase (www.mirbase.org), up to 10 different miRNA399 species (miR399a to j) were identified in the *M. truncatula* genome. Sequence alignment distinguished up to 5 different variants of miR399 **(** **Supplementary Figure 2B** **)** that potentially could target the miR399BS identified within the 5′-UTR of the *MtPHO2* genes. Depending on the miR399BS, mismatches were identified in the central or toward the 3′ ends of the miR399s sequences.

The *MtPHO2-A* gene was predicted to have three splicing isoforms **(** **Supplementary Figure 1A** **)**. Amino acid sequence alignment and phylogenetic analysis of the 81 PHO2 proteins identified in the sequenced plant genomes revealed phylogenetic patterns, grouping into clades. Except for the Poales order, most of the PHO2 identified in the databases fell into different plant orders of Eudicots. Gene duplication events were identified in monocots and eudicots, with close evolutionary relationships (92-98% homology). In legumes (order Fabales), two distinct branches associated with the well-known genome duplication event (Cannon et al., 2006) were identified. MtPHO2-A and MtPHO2-B appeared clustered together showing 84% homology at the protein level, while MtPHO2-C clustered in a duplicated branch **(** **Figure 1B** **)** sharing 68% homology with MtPHO2-B and 60% with MtPHO2-A (data not shown).

A detailed analysis revealed that four of the five possible MtPHO2 protein variants (including the three MtPHO2-A variants) conserve the distinctive ubiquitin-conjugating catalytic (UBCc) domain at the C-terminus, including E3 ligase interaction residues and the E2 active site cysteine according to PROSITE database (https://prosite.expasy.org/). MtPHO2-A.3 protein variant was the only exception, lacking the UBCc domain **(** **Supplementary Figure 1B** **)**.

### *MtPHO2* expression is regulated by P and N availability

**Figure 2:**
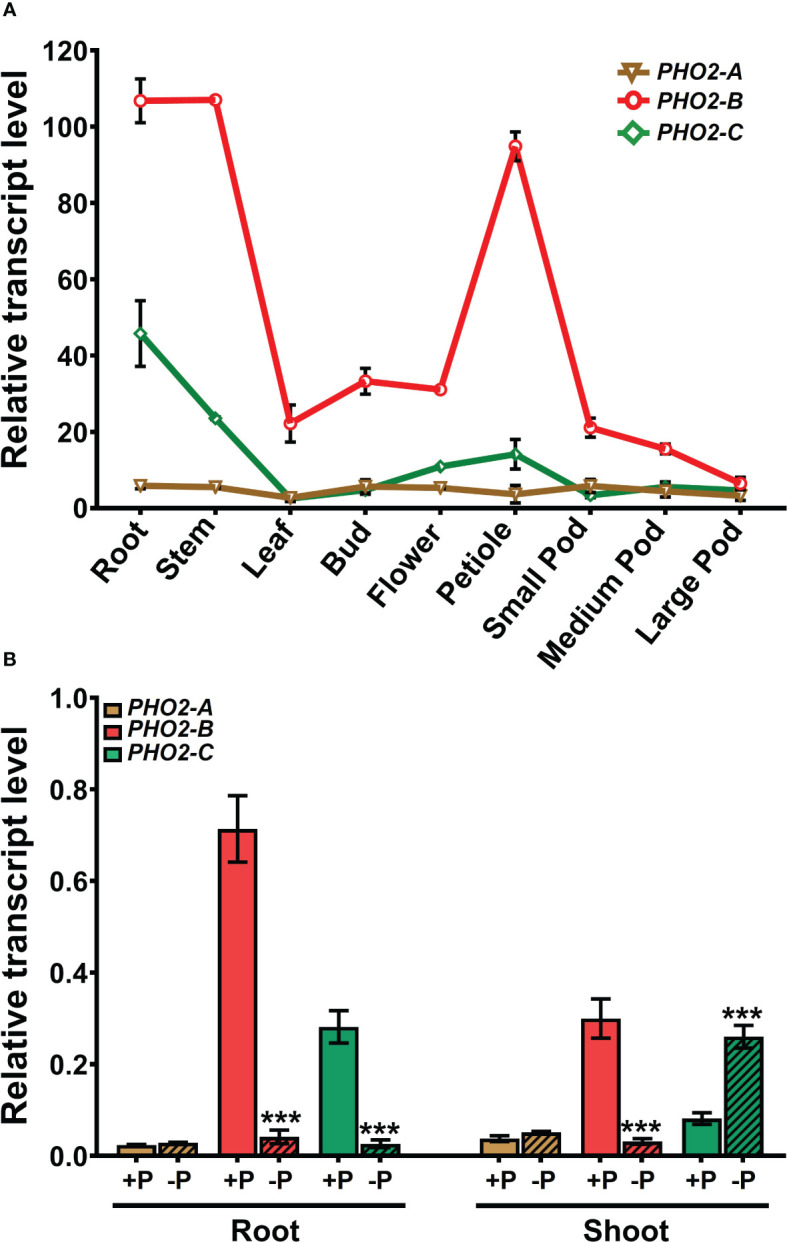
Expression profiles of *Medicago truncatula PHO2*-like genes in various organs and treatments.**(A)** Relative transcript levels of *MtPHO2-A*, *B*, *C* in different organs derived from RNA-seq data with numbers indicating fragments per kilobase of transcript per million reads mapped (FPKM) and represent the average of three biological replicates with standard errors. Further details are given in Boschiero et al. (2020) and the *M. truncatula* SSP database (MtSSPdb). **(B)** Relative transcript levels of *MtPHO2-A*, *B* and *C*, determined by qRT-PCR analysis of roots and shoots of four-week-old plants grown under optimal P-nutrition (+P, 0.5 mM Pi) and limiting-P (-P, 20 μM Pi) conditions, as shown in **Figure 4** and as described in the methodology. PCR primer sequences are presented in **Supplementary Table S2**. Data shown are the mean and SEM of three independent experiments (n=6). For each replicate, transcript levels were normalized against two housekeeping genes, *MtPTB2* and *MtPDF2*; asterisks indicate significant differences between the optimal and reduced P (*p < 0.05, **p < 0.01, ***p < 0.001) calculated using two-tailed Student’s t-tests.

**Figure 3:**
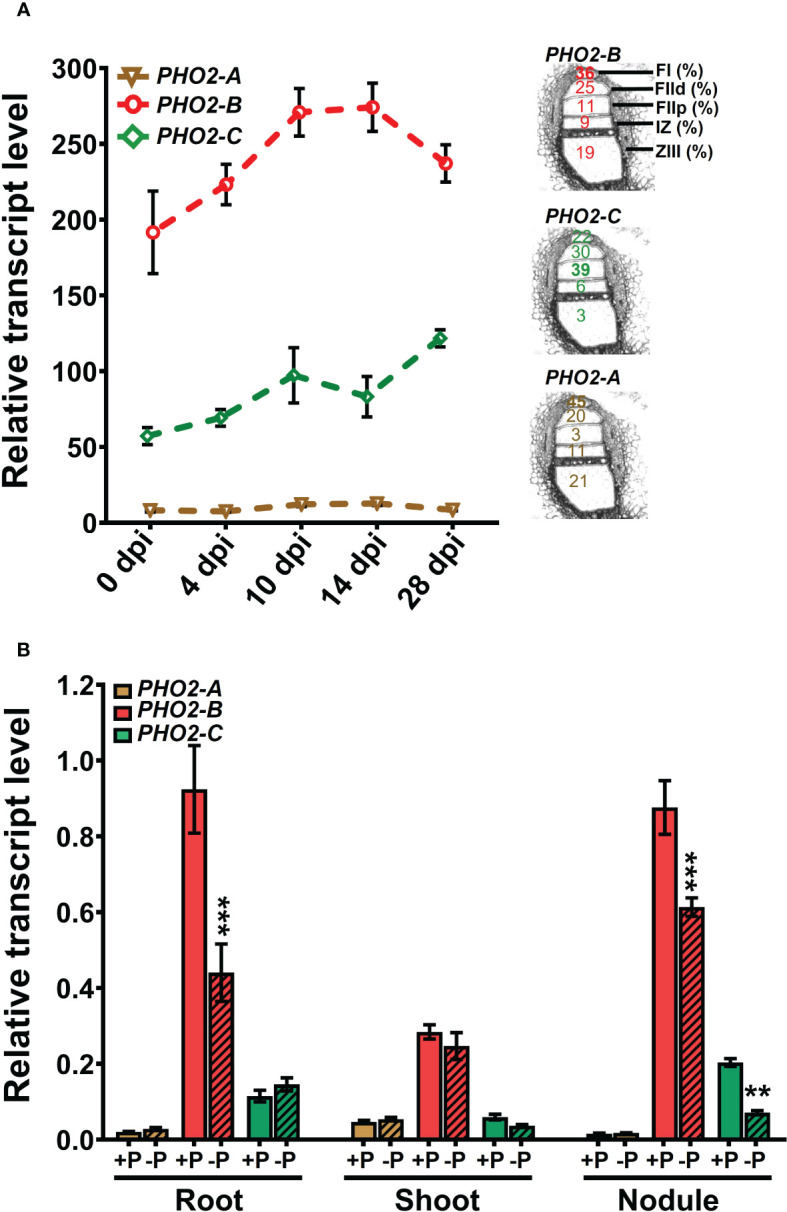
Expression profiles of *Medicago truncatula PHO2* genes in various organs and treatments during nodulation. **(A)** Relative transcript levels of *MtPHO2-A*, *B* and *C* in nodules over time. Left, RNA-seq data expressed as fragments per kilobase of transcript per million reads mapped (FPKM) represent the average of three biological replicates with standard errors. Further details are given in the MtSSPdb (Boschiero et al. (2020). Right: scheme of the five laser micro-dissected regions of *M. truncatula* nodules, containing the percentage of normalized counts per region for each *PHO2* gene, according to Roux et al. (2014). **(B)** Relative transcript levels, quantified by qRT-PCR, of *MtPHO2-A*, *B* and *C* genes in four-week-old roots, shoots and nodules of plants grown under symbiotic nitrogen fixation conditions (0.5 mM N and inoculated with *S. meliloti* strain Sm1021) with optimal-P (+P, 0.5 mM P) or reduced-P (-P, 20 μM P), under the same conditions as those shown in **Figures 5**–**8**. PCR primer sequences are presented in **Supplementary Table 2**. Data are the mean and SEM of three independent experiments (n=6 for root and shoot, n=18 for nodules). For each replicate, transcript levels were normalized against two housekeeping genes (*MtPTB2* and *MtPDF2*); asterisks indicate significant differences between optimal- and reduced-P, calculated using two-tailed Student’s t-tests (*p < 0.05, **p < 0.01, ***p < 0.001).

We investigated the spatial-temporal expression patterns of *MtPHO2* genes. Relative transcript levels, based on sequence fragments per kilobase of transcript per million reads mapped (FPKM) (MtSSPdb; https://mtsspdb.zhaolab.org/database), revealed that *MtPHO2* genes were ubiquitously expressed in different organs and pod developmental stages, although their expression was consistently higher in the root. *MtPHO2-B* had the highest expression level in all the organs evaluated, followed by *MtPHO2-C. MtPHO2-A* expression was relatively low in all organs **(** **Figure 2A** **)**. Similar expression differences between these genes were detected during nodulation, with only slight changes between 10- and 28-days post inoculation (dpi). Again, *MtPHO2-A* expression levels were very low compared to the other two *MtPHO2* genes **(** **Figure 3A** **)**.

Expression profiles determined by qPCR confirmed that in the absence of nutritional deficits, *MtPHO2-B* was the primary *PHO2* transcript in roots and shoots, followed by *MtPHO2-C* and *MtPHO2-A* **(** **Figure 2B** **)**. P-limitation resulted in lower transcript levels of *MtPHO2-B* and *MtPHO2-C* in roots. In shoots, only *MtPHO2-B* was down-regulated under P-limitation while *MtPHO2-C* was up-regulated **(** **Figure 2B**; **Supplementary Figure 3A** **)**. N-limitation resulted in up-regulation of *MtPHO2-B* and *MtPHO2-C* in roots and shoots, but no significant change in *MtPHO2-A* transcript levels **(** **Supplementary Figure 3B** **)**.

Expression profiles of the three *MtPHO2* genes in symbiotic, nitrogen-fixing plants were similar to those of non-symbiotic plants, with *MtPHO2-B* exhibiting the highest transcript levels in roots, shoots, and nodules, followed by *MtPHO2-C* and *MtPHO2-A* **(** **Figure 3B**; **Supplementary Figure 3A** **)**. P-limitation under symbiotic conditions down-regulated *MtPHO2-B* in roots and nodules but not shoots. *MtPHO2-B* and *MtPHO2-C*, but not *MtPHO2-A*, were down-regulated in nodules in response to P-limitation **(** **Figure 3B** **)**.

### Functional characterization of the *MtPHO2* genes under optimal, non-symbiotic conditions

**Figure 4:**
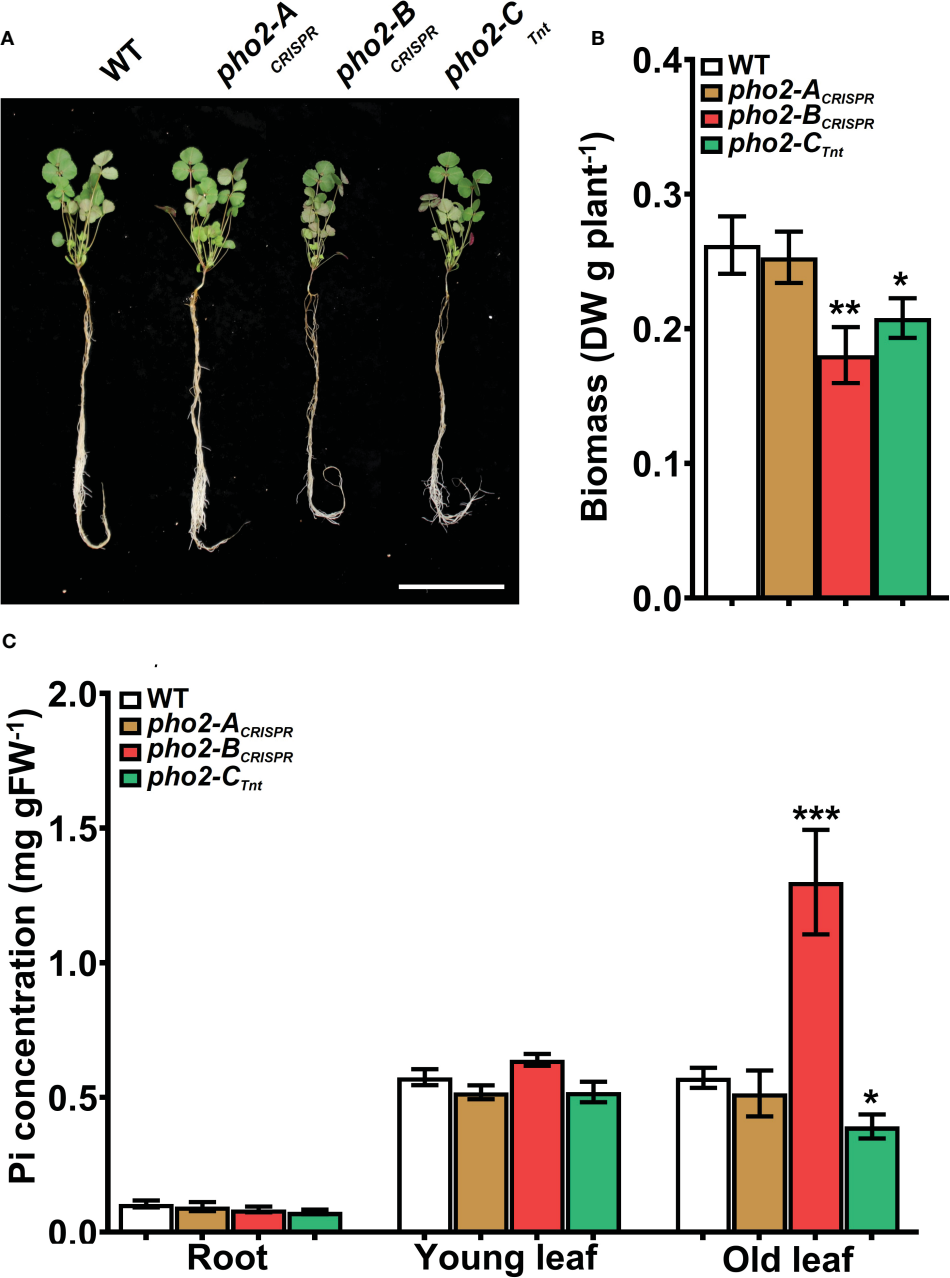
Performance of the *pho2* mutants under optimal nutritional conditions. **(A)** Representative four-week-old plants growth under optimal nutritional conditions, including 0.5 mM Pi. Scale bar = 10 cm. **(B)** Plant dry weight. **(C)** Free phosphate (Pi) concentration in roots, young and old leaves. Data shown are the mean and SEM of three independent experiments (n=5 per experiment). Asterisks indicate significant differences between the wild type and the mutants calculated using two-tailed Student’s t-tests (*p < 0.05, **p < 0.01, ***p < 0.001).

To explore the function of the MtPHO2 proteins, homozygous *pho2-A*_CRISPR_, *pho2-B*_CRISPR_, *pho2-A*_Tnt1_, *pho2-B*_TALEN_ and *pho2-C*_Tnt1_ mutants were used **(** **Supplementary Figure 4** **).** Under optimal, nutrient-replete conditions, lack of MtPHO2-B or MtPHO2-C reduced root and shoot growth, especially in the case of the *pho2-B*_CRISPR_ mutant **(** **Figures 4A, B** **)**. Measurement of Pi accumulation in roots, young leaves and fully expanded mature leaves revealed that only the *pho2-B*_CRISPR_ mutant had significantly higher Pi accumulation in mature leaves, compared to the WT, while the *pho2-C*_Tnt1_ mutant showed a slight but significant reduction in Pi content in such leaves **(** **Figure 4C** **)**. Plants of each mutant line were also grown in rich soil (Metro-Mix) in a greenhouse under optimal nutritional conditions for analysis of late developmental phenotypes and seed replication. All three mutants grew less than WT plants, although the *pho2-B*_CRISPR_ mutant was the most severely affected, followed by *pho2-C*_Tnt1_ and *pho2-A*_CRISPR_ mutants **(** **Supplementary Figure 5** **)**. Stunted growth was accompanied by a decrease in seed production in both the *pho2-B*_CRISPR_ and *pho2-C*_Tnt1_ mutants, especially the former, which also displayed symptoms of necrosis in its mature leaves **(** **Supplementary Figure 5** **)**.

### Functional characterization of MtPHO2 genes under symbiotic nitrogen fixation conditions

**Figure 5:**
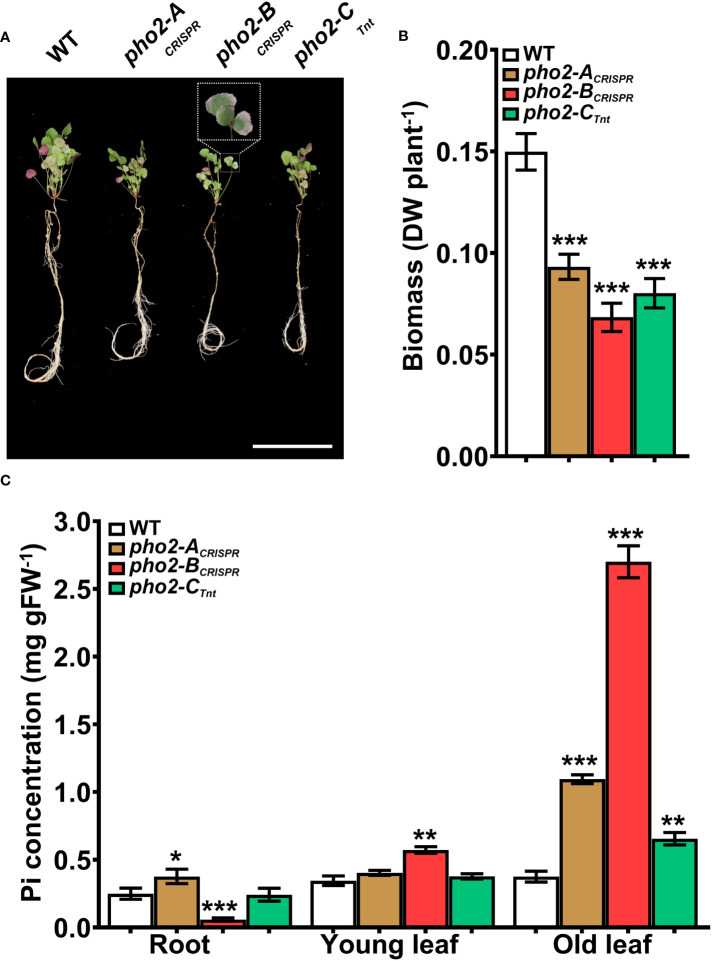
Performance of the Mt*pho2* mutants under symbiotic nitrogen fixation conditions. Plants were inoculated with *S. meliloti* strain Sm1021 and given optimal-P (0.5 mM Pi). **(A)** Representative four-week-old plants. Scale bar = 10 cm. **(B)** Plant dry weight. **(C)** Free phosphate (Pi) concentration in roots, young and old leaves. Data shown are the mean and SEM of three independent experiments (n=5/experiment). Asterisks indicate significant differences between the wild type and the mutants calculated using two-tailed Student’s t-tests (*p < 0.05, **p < 0.01, ***p < 0.001).

All three mutants, *pho2-A*_CRISPR_, *pho2-B*_CRISPR_ and *pho2-C*_Tnt1_ exhibited reduced growth and biomass under optimal symbiotic conditions, including high Pi (0.5 mM; **Figure 5**). Again, mature leaves of the *pho2-B*_CRISPR_ mutant, but not the other mutants, exhibited necrotic symptoms **(** **Figures 5A, B** **)**. Measurements of organ Pi content revealed hyper-accumulation of Pi in young and especially mature leaves of the *pho2-B*_CRISPR_, which was mirrored by a drastic decrease in Pi content of its roots. Pi also accumulated in older leaves of both the *pho2-A*_CRISPR_ and *pho2-C*_Tnt1_ mutants relative to the WT **(** **Figure 5C** **)**.

**Figure 6:**
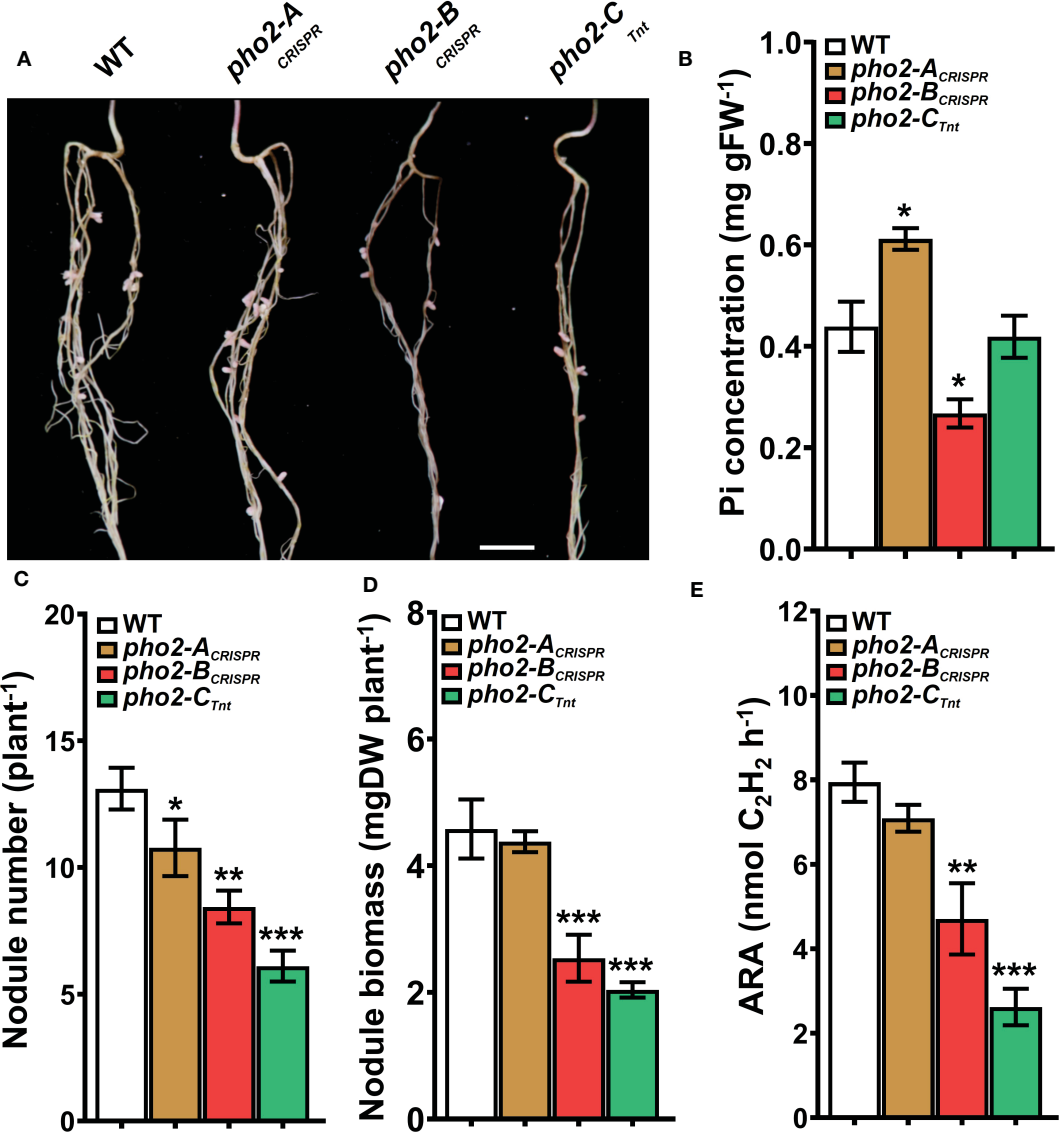
Symbiotic phenotypes of the Mt*pho2* mutants with optimal-P. **(A)** Nodulated roots at 21 dpi with *S. meliloti* strain Sm1021. Scale bar = 1 cm. **(B)** Free phosphate (Pi) concentration in nodules **(C)** Average nodule number. **(D)** Average nodule biomass. **E)** Acetylene reduction assay (ARA) on whole nodulated roots. Data shown are the mean and SEM of three independent experiments (n=5/experiment). Asterisks indicate significant differences between the wild type and mutants calculated using two-tailed Student’s t-tests (*p < 0.05, **p < 0.01, ***p < 0.001).

Traits related to SNF were differentially affected in the three mutants. The *pho2-A*_CRISPR_ mutant showed a reduced number of nodules compared to the WT, but with similar biomass and nitrogenase activity **(** **Figures 6A-E** **)**. The *pho2-B*_CRISPR_ mutant was affected in all the traits evaluated, with reduced number and biomass of nodules, as well as nitrogen fixation capacity. Likewise, *pho2-C*_Tnt1_ exhibited reduced nodule number, biomass and nitrogen fixation **(** **Figures 6A-E** **)**. Loss of gene function had a variable effect on nodule Pi concentration, with the *pho2-B*_CRISPR_ accumulating less, *pho2-A*_CRISPR_ accumulating more, and *pho2-C*_Tnt1_ accumulating the same concentration as the WT **(** **Figure 6B** **)**.

**Figure 7:**
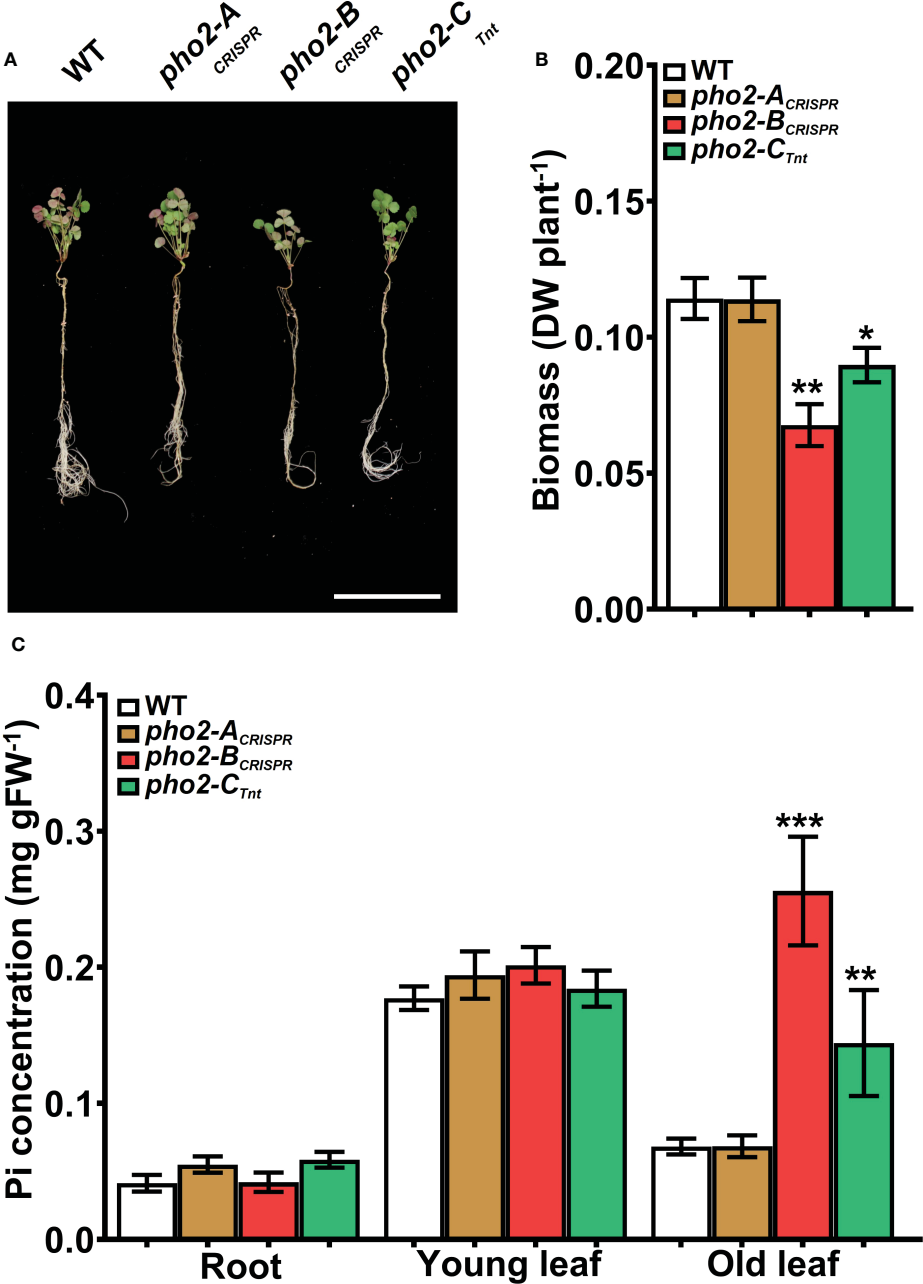
Performance of the *Mtpho2* mutants under symbiotic nitrogen fixation conditions. Plants were inoculated with *S. meliloti* strain Sm1021 and given reduced-P (20 μM Pi). **(A)** Representative four-week-old plants. Scale bar = 10 cm. **(B)** Plant dry weight. **(C)** Free phosphate (Pi) concentration in roots, young and old leaves. Data shown are the mean and SEM of three independent experiments (n=5/experiment). Asterisks indicate significant differences between the wild type and the mutants calculated using two-tailed Student’s t-tests (*p < 0.05, **p < 0.01, ***p < 0.001).

**Figure 8:**
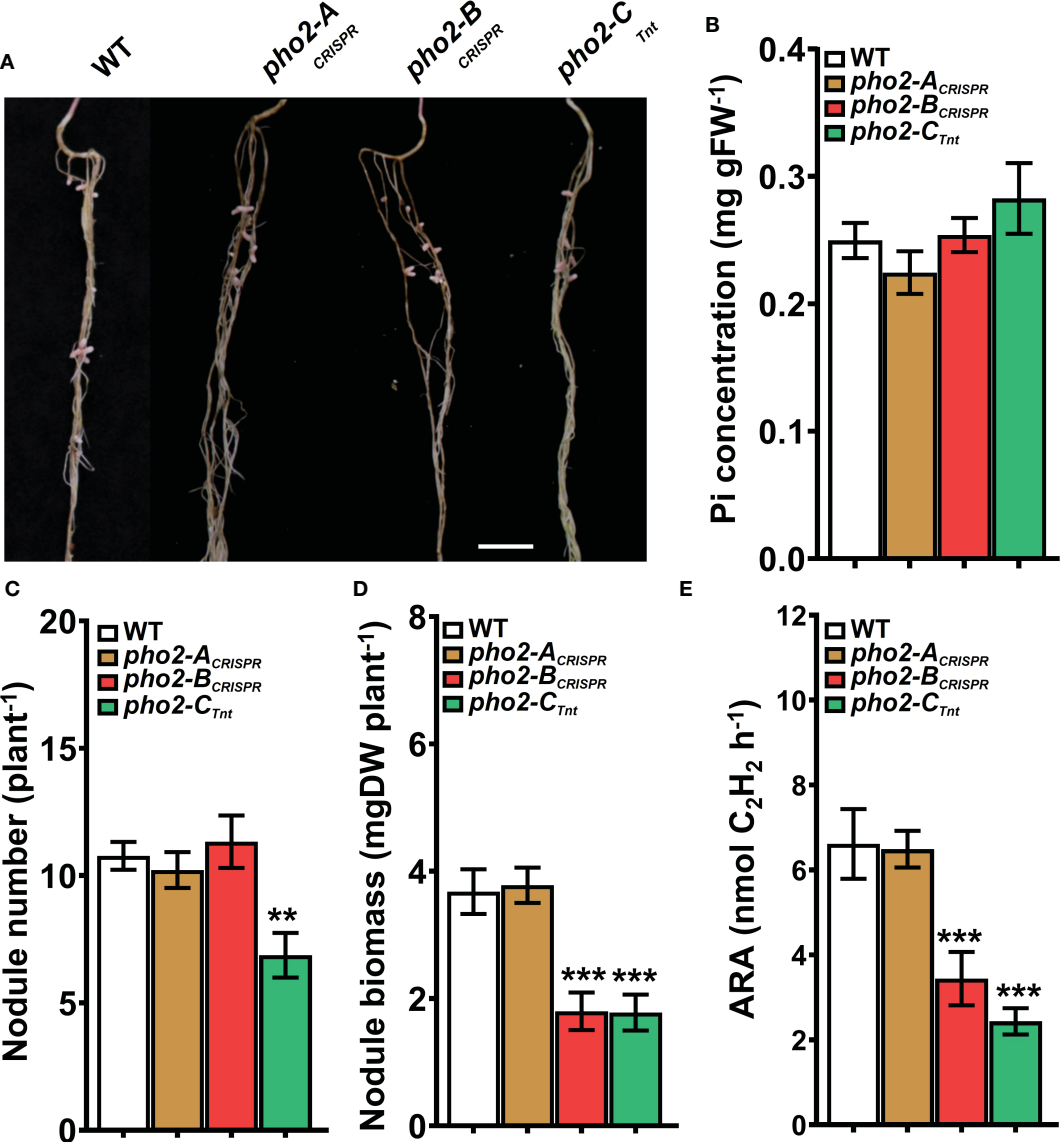
Symbiotic phenotypes of the *Mtpho2* mutants with reduced-P. **(A)** Nodulated roots at 21 dpi with *S. meliloti* strain Sm1021. Scale bar = 1 cm. **(B)** Free phosphate (Pi) concentration in nodules **(C)** Average nodule number. **(D)** Average nodule biomass. **(E)** Acetylene reduction activity (ARA) of whole nodulated roots. Data shown are the mean and SEM of three independent experiments (n=5/experiment). Asterisks indicate significant differences between the wild type and mutants calculated using two-tailed Student’s t-tests (*p < 0.05, **p < 0.01, ***p < 0.001).

P-limitation reduced WT plant growth, biomass, nodule number and biomass, nitrogen fixation, and Pi concentration in all organs relative to P-replete plants (compare **Figures 7**, **8** with **Figures 5**, **6**, respectively). Even so, *pho2-B*_CRISPR_ and *pho2-C*_Tnt1_ mutants were smaller than the WT under P-limiting conditions **(** **Figures 7A, B** **)**. The reduced size of these two mutants was accompanied by a moderate but significant accumulation of Pi in mature leaves compared to the WT **(** **Figure 7C** **)**. *pho2-B*_CRISPR_ and *pho2-C*_Tnt1_ mutants also exhibited defects in symbiotic traits, including reduced nodule biomass and nitrogen fixation **(** **Figures 8C-E** **)**. Interestingly, reduced nodule biomass resulted from reduced nodule number of *pho2-C*_Tnt1_ but not *pho2-B*_CRISPR,_ which produced the same number of nodules as the wild-type, albeit smaller **(** **Figure 8** **)**.

### Transcriptomic responses associated with Pi content modifications

**Figure 9:**
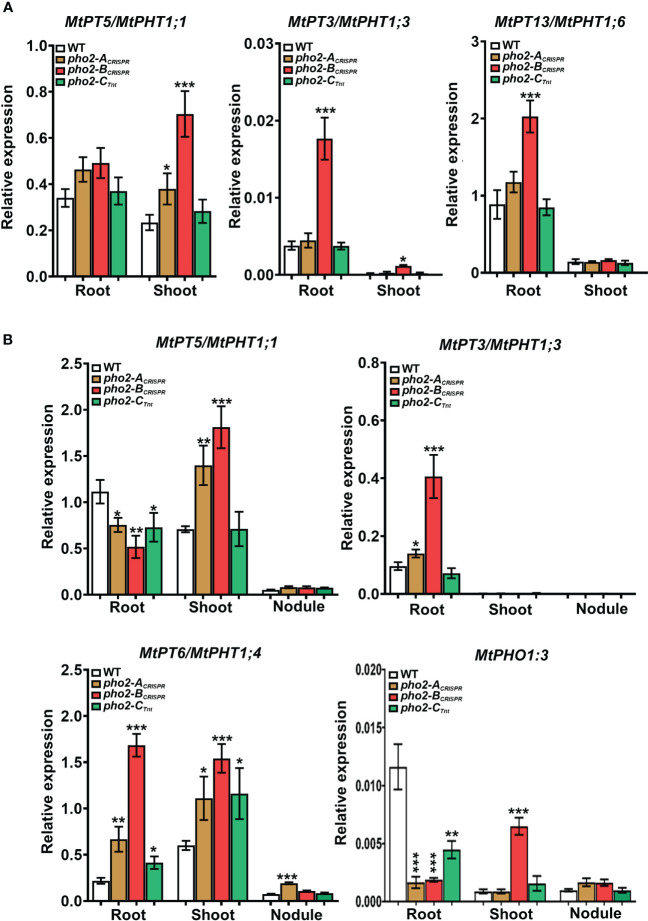
Relative expression levels, quantified by qPCR, of *MtPHT1-like* genes in various experimental conditions and organs **(A)** Optimal-P. **(B)** Symbiotic nitrogen fixation with optimal-P. PCR primers used are included in **Supplementary Table S2**. Data shown are the mean and SEM of three independent experiments. Plants were grown under the same conditions as those shown on **Figures 4**–**8**. For each replica, copy numbers were normalized using the mean average of two housekeeping genes (MtPTB2 and MtPDF2); asterisks indicate significant differences between the WT and mutants in each tissue of the same condition calculated using two-tailed Student’s t-tests (*p < 0.05, **p < 0.01, ***p < 0.001). Double nomenclature (PT/PHT) due to Breuillin-Sessoms et al., 2015. Plant Cell. 27(4):1352-66.999.

To better understand the role of PHO2 homologs in Pi homeostasis, we measured the expression levels of eight *MtPT*/*PHT1-like*, five *MtPHO1-like*, three nitrogen limitation adaptation (NLA) and three PHR1-like genes in roots and shoots of WT and mutant plants **(** **Supplementary Table 2** **)**. Quantification of expression levels by qPCR revealed that all *MtPT*/*PHT1-like* and *MtPHO1-like* transporters were induced by P-limitation (data not shown). In the absence of nutritional deficits, *MtPT5*, *MtPT3* and *MtPT13* transporters were highly upregulated in roots and/or shoots of *pho2-B*_CRISPR_ plants compared to the WT plants, whereas *MtPT5* was slightly upregulated in shoots of *pho2-A*_CRISPR_ plants **(** **Figure 9A** **)**. Under symbiotic conditions without P limitation, the three mutant alleles presented deregulation of the expression levels of *MtPT*/*PHT1-like* transporters. The expression levels of *MtPT5*, *MtPT3*, *MtPT6* and *MtPHO1;3* transporters were deregulated in roots and/or shoots of *pho2-B*_CRISPR_ plants compared to the WT plants. These transporters, except for *MtPHO1;3*, also showed deregulation in their expression levels in *pho2-A*_CRISPR_ plants, but more moderate than those in *pho2-A*_CRISPR_ plants. Similarly, *MtPT5*, *MtPT6* and *MtPHO1;3* transporters were deregulated in roots and/or shoots of *pho2-C*_CRISPR_ plants compared to the WT plants. Although *MtPT6* was upregulated at different levels in roots and shoots of all the three mutants, it was the only transporter upregulated in the nodule of the *pho2-A*_CRISPR_ mutant compared with the WT plants **(** **Figure 9B** **)**.

## Discussion

*PHOSPHATE2* (*PHO2*) genes encode PHO2/Ubiquitin-Conjugating E2 24 (UBC24) proteins. The role of PHO2 proteins in regulating inorganic phosphate (Pi) homeostasis and Pi translocation and remobilization has been illuminated using mutants of Arabidopsis (Bari et al., 2006), rice (Cao et al., 2014), common wheat (Ouyang et al., 2016), and alfalfa (Miller et al., 2022), and transient silencing in barley (Pacak et al., 2010; Pacak et al., 2016) and citrus (Wang et al., 2020). The regulatory role of PHO2 in Pi homeostasis is based on its joint action with E3 ligases to polyubiquitinate and degrade Pi transporters, thus controlling Pi translocation, accumulation and/or remobilization in plant tissues when P is not limiting. Under P limitation, turnover of Pi transporters is prevented by undermining PHO2 protein production *via* microRNA399 (miR399)-guided*PHO2* mRNA degradation, orchestrated by the transcription factor PHOSPHATE STARVATION RESPONSE 1 (PHR1) (Aung et al., 2006; Bari et al., 2006).

Although some species, e.g. Arabidopsis and rice (Bari et al., 2006; Cao et al., 2014), have a single *PHO2* gene, others have more than one, which begs the question of their roles in P-homoeostasis or other plant processes. For instance, autotetraploid alfalfa has two *PHO2* genes, with multiple haplotypes of each. However, only one of the two alfalfa PHO2 proteins seems to have a role in P-homeostasis (Miller et al., 2022).

Here, we used sequence and phylogenomic analysis to identify three different *PHO2* genes in the *Medicago truncatula*, encoding up to five possible PHO2 proteins, including splice variants **(** **Figure 1** **;** **Supplementary Figure 1** **)**. In addition to having three *PHO2* genes, the presence of splicing isoforms adds an extra level of complexity that may have biological significance. Alternate splicing to produce different functional proteins is a tightly regulated process essential for plants development and adaptation to environment (Huertas et al., 2019). In fact, *PHO2* transcript splicing is regulated by P stress in rice (Secco et al., 2013), while Arabidopsis produces a shorter spliced form of its single *PHO2* gene transcript that does not contain miR399 binding sites, to escape from miR399 mediated target degradation (Scheible et al., 2023). Two splice variants of different length have been reported for *PHO2* in barley (Pacak et al., 2016) but have not been characterized. Interestingly, detailed study of splicing events in tomato under P stress revealed none in *SlPHO2* (Tian et al., 2021), suggesting that alternative splicing of *PHO2* transcript may not occur in all species and/or conditions. It is interesting that one of the Medicago splice variants, Mt*PHO2-A.3*, which lacks its UBC catalytic domain sequences **(** **Supplementary Figure 1** **)**, has a counterpart in rice (Dong et al., 2018). Further work is required to understand what role, if any, such a protein plays in Medicago and rice.

Transcript expression levels of the *PHO2* genes are modulated by nutritional status, being normally repressed in roots and shoots by P limitation (Bari et al., 2006; Hackenberg et al., 2013; Cao et al., 2014; Ouyang et al., 2016; Wang et al., 2017; Miller et al., 2022; Santoro et al., 2022). Recent reports in Arabidopsis, tomato and barley revealed that *PHO2* genes also can alter their expression as a function of N levels, indicating their integrative role between P and N homeostasis (de Souza Campos et al., 2019; Medici et al., 2019; Marro et al., 2022). Remarkably, some *PHO2* genes, however, have different time-, intensity-, and genotype-dependent transcriptomic responses under P limitation (de Souza Campos et al., 2019; Gamir et al., 2020), indicating complex spatial-temporal Pi starvation signaling. Modulation of *PHO2* transcripts requires recognition of *PHO*2 mRNAs by miR399s, regulated in turn by the transcription factor PHR1. Thus, the presence of five different miR399-BS conserved in the 5′-UTR of the three *MtPHO2* genes underscores their likely role in P regulation **(** **Figure 1A**; **Supplementary Figure 2** **)**. The high identity between the 10 *Medicago truncatula* miR399s and miR399-BSs on their 5′-UTR **(** **Supplementary Figure 2** **)** suggests their potential to cleave *MtPHO2* mRNAs. Although we did not validate miR399 cleavage, the high homology of the Medicago miR399 and miR399-BS sequences with those of other plant species already validated *in vitro* (Aung et al., 2006; Bari et al., 2006; Hackenberg et al., 2013; Xu et al., 2013; Miller et al., 2022) or by transient expression (Ouyang et al., 2016), strongly suggest their cleavage capabilities. Indeed, regulation of expression of *MtPHO2-B* by a miR399 has already demonstrated indirectly through the mutation of the Medicago *PDIL1* (Wang et al., 2017), a Pi deficiency-responsive At4-like lncRNA that suppresses the effect of miR399 by acting as mimics of PHO2 (Franco-Zorrilla et al., 2007). Interestingly, *MtPHO2-B* gene also contain a PHR1-binding DNA sequence (P1BS) motif. To our knowledge, this is the first description of the P1BS motif in a *PHO2* gene, suggesting direct PHR1-dependent regulation in addition of the mediated regulation by the various miR399s. The presence of miR399s variants and cis-regulatory elements **(** **Figure 1A** **;** **Supplementary Figure 2** **)** could lead to different post-transcriptional regulations that could explain the different levels of expression and/or transcriptomic responses between the *MtPHO2-B* and *MtPHO2-C* genes to the P nutritional status in the plant **(** **Figures 2**, **4** **)**, suggesting then a differential role for both genes. Likewise, Mt*PHO2-B*, and to a much lesser extent *MtPHO2-C*, are induced by N limitation in roots and shoots **(** **Supplementary Figure 3** **)**, suggesting a similar regulation dependent on the N status of the plant.

The impaired growth observed under control conditions by the lack of the protein MtPHO2-B can directly be associated with un unbalanced Pi homeostasis due to Pi hyperaccumulation in old mature leaves **(** **Figure 4** **)**. This is the result of a deficiency PHO2-dependent degradation of Pi transporters, altering Pi movement between tissues and organs and preventing Pi from being used properly by the plant (Huang et al., 2013; Park et al., 2014). Taken together, our results identify MtPHO2-B as the functional ortholog of the characterized PHO2 proteins (Aung et al., 2006; Bari et al., 2006; Cao et al., 2014; Ouyang et al., 2016; Miller et al., 2022), integral to Pi homeostasis in the absence of nutritional deficits. Similarly, the reduced plant growth in the absence of the protein MtPHO2-C can also be associated with an imbalanced P homeostasis. However, since *pho2* mutants are described as Pi hyperaccumulators (Aung et al., 2006; Bari et al., 2006) and the *M. truncatula* mutant has a marginal, albeit significant, reduction in Pi content in old leaves **(** **Figure 4** **)**, our results implies that *MtPHO2-C* is not a functional ortholog of the characterized PHO2, although it does appear to have a role in Pi homeostasis. The proposed secondary role controlling Pi homeostasis is in line with results recently described for the alfalfa homologous *PHO2-C*, where mutations of the different haplotypes lead to limited Pi hyperaccumulation possibility because to the inability of miR399s to cleave *MtPHO2-C* mRNAs (Miller et al., 2022). Demonstrating the role of the MtPHO2-C proteins in Pi homeostasis, regardless of any putative role in plant development **(** **Supplementary Figure 5** **)**, will require further experiments. Likewise, the reduced expression levels and lack of transcriptomic response to P limitation suggest a limited role in controlling Pi homeostasis, or compensation by the other two MtPHO2 proteins. This is partially supported by the absence of phenotypes in the mutant alleles during the early stages of development but does not rule out a long-term role in regulating certain levels of Pi necessary to support plant growth **(** **Supplementary Figure 5** **)**. In fact, *Medicago truncatula* is more sensitive to elevated Pi content compared with most other leguminous and non-leguminous plant species (Sulieman et al., 2013), so it is possible to reason that it also requires a finely tuned Pi homeostasis, controlled by multiple PHO2 activities.

Symbiotic nitrogen fixation (SNF) is a complex series of physical and chemical interactions built upon the trading of reduced carbon (C) from a legume for reduced N from the compatible symbiont. Atmospheric nitrogen (N_2_), once fixed in the root nodules, is partially transported to the aerial parts of the plant supporting multiple metabolic processes (Roy et al., 2020). The intensive C turnover during nodule development and N_2_ fixation are energy-consuming processes, requiring a large amount of P (Cabeza et al., 2014), as well as a limited presence of mineral N to promote nodule development without actually inhibiting N_2_ fixation (Ferguson et al., 2019). Under this condition of mineral N deprivation, most of the N available for plant use is obtained from the N_2_ fixation, so plants need a fine adjustment of Pi homeostasis in the nodule coordinated with the whole-plant Pi homeostasis (Sulieman & Tran, 2015; Sulieman et al., 2019). Such level of coordination in *Medicago truncatula* seems to be provided by the three MtPHO2 proteins, although the protein PHO2-B has a leading role in maintaining Pi homeostasis, as evidenced by the huge Pi hyperaccumulation not only in old leaves but also in young leaves, while it locally disappears from the roots **(** **Figure 5C** **)** and nodules **(** **Figure 6B** **)** in the *pho2-B* mutant. To our knowledge, this is the first time that a *pho2* mutant imbalances Pi throughout the whole plant, including nodules. The abnormal distribution of Pi impacts nodule formation and development, causing the reduction in N_2_ fixation activity **(** **Figures 6C, D** **)** (Hernandez et al., 2009), impairing plant growth **(** **Figures 5A, B** **).** Thus, although plants preferentially distribute available Pi to the nodules to maintain symbiotic N_2_ fixation (Cabeza et al., 2014; Sulieman & Tran, 2015), our results confirm that nodule Pi homeostasis is subordinated to the whole-plant Pi homeostasis. Nevertheless, Pi homeostasis under SNF is not regulated by a single *MtPHO2* gene. Even if at a lower scale than MtPHO2-B, the proteins MtPHO2-C and MtPHO2-A are also necessary to maintain whole-plant Pi homeostasis, the latter controlling Pi accumulation in roots and old leaves **(** **Figure 5** **)**, and specially regulating Pi content for the formation of nodules **(** **Figures 6B, D** **)**. The fact that neither the diploid nor the tetraploid alfalfa genome contains an orthologous *MtPHO2-A* gene while it is present in the wild relative *Medicago ruthenica* (Wang et al., 2021; Miller et al., 2022) and in *Medicago truncatula* genomes, evidence the evolutionary importance of this gene controlling quantitative variation for nodule formation (Stanton-Geddes et al., 2013; Curtin et al., 2017). Therefore, we can speculate that MtPHO2-A gene diverged from MtPHO2-B **(** **Figure 1B** **)** to support a finely tuned Pi homeostasis, especially in root nodules.

Symbiotic nitrogen fixation under limited mineral P is severely impacted by reducing the formation and the development of nodules, reducing the fixed N_2_ to be used by the plant **(** **Figure 8** **)** (Sulieman et al., 2013). In these conditions, MtPHO2-A protein does not seem to play a relevant role in the whole-plant Pi homeostasis while MtPHO2-B and MtPHO2-C proteins continue to exert some Pi regulatory capacity **(** **Figure 7** **)**, necessary to regulate SNF traits **(** **Figure 8** **)**. This, which could be somewhat unexpected since the role of PHO2 proteins, associated with the regulation of Pi when it is in excess, is necessary to coordinate N and P homeostasis (Liese et al., 2017; Medici et al., 2019) of the nodules to use Pi efficiently according to N availability. However, the reduced SNF due to P limitation also involves a lower degree of N_2_ assimilation, and therefore it is expected that plants exhibit local and/or systemic N-limited-related responses. As mentioned earliest, *MtPHO2-B* and *MtPHO2-C* genes are both induced by N deficiency **(** **Supplementary Figure 3** **)**, but their tissue-dependent transcriptomic responses to mineral N and P limitation suggest common functions within the nodule, but also some type of speciation between tissues **(** **Figure 3B** **)**. In tomato, for example, Pi starvation signaling depends on the plant’s N status, such that the downregulation of *SlPHO2* by Pi deficiency happens only under optimal N conditions, whereas it can be upregulated by N deficiency only under optimal P conditions (Marro et al., 2022). Our results point here, indicating that both proteins MtPHO2-B and MtPHO2-C have a similar role, coordinating whole-plant P and N homeostasis to sustain plant growth.

Overall, our results confirm that under mineral N deprivation, the fine adjustment of Pi homeostasis is primarily due to the integral action of MtPHO2-B, assisted by the action of MtPHO2-C and MtPHO2-A. The latter also seems to have a specific role regulating nodule Pi homeostasis associated to nodule formation.

Tissue hyperaccumulation of Pi in *pho2* mutants is known to result in local Pi limitation in other plant tissues and organs. To compensate for this local Pi limitation, these plants alter the expression levels of PHO1-like and PT/PHT1-like transporters, components of the P starvation response (PSR) (Aung et al., 2006; Bari et al., 2006; Hu et al., 2011; Liu et al., 2012; Huang et al., 2013; Ouyang et al., 2016). In fact, some of the PT/PHT1 transporters targeted by the PHO2-E3 complex to be degraded can increase their mRNA levels in the pho2 mutants (Huang et al., 2013; Lin et al., 2013). Under non-limiting nutritional conditions, only the lack of MtPHO2-B protein resulted in a higher expression of MtPT5, MtPT3 and MtPT13 in roots and/or shoots compared to WT **(** **Figure 9A** **)**, affirming that PHO2-B is the main regulator of Pi homeostasis in Medicago truncatula. Although it is not possible to know if these changes are directly or indirectly linked to MtPHO2-B activity, the fact that there are no differences in the others individual mutants, points at these Pi transporters as PHO2-E3 targets for future experiments in *Medicago truncatula*.

The different patterns of Pi hyperaccumulation also lead to transcriptomic responses of the PSR components during SNF. Interestingly, although the genes *MtPHO2-B* and *MtPHO2-C* are the more abundant *MtPHO2* genes within the nodules **(** **Figure 3** **)** (Roux et al., 2014), the lack of these individual proteins did not alter the expression of any of the Mt*PHO1-like* and Mt*PT/PHT1-like* genes in the nodules, but in roots and/or shoots **(** **Figure 9B** **)**, supporting their role in maintaining whole-plant Pi homeostasis instead of nodule Pi homeostasis. These results contrast with the fact that some of the *PHO1-like* and *PT/PHT1-like* transporters, expressed between the root vascular system and vascular bundles of nodules, are crucial to maintain the Pi reallocation and homeostasis in young and mature nodules (Qin et al., 2012; Chen et al., 2019; Lu et al., 2020; Nguyen et al., 2021). On the contrary, the lack of the protein MtPHO2-A altered the expression of Mt*PT6/PHT1;4* in nodules **(** **Figure 9B** **)**, one of the two *PT/PHT1-like* transporters that are induced during nodulation according to the MtSSPdb (Boschiero et al., 2020; https://mtsspdb.zhaolab.org/atlas-internal), which support the unique role of MtPHO2-A in maintaining Pi homeostasis in the nodule. New studies are needed to determine the role of MtPT6-like in SNF and the different variants of the MtPHO2-A protein, and a much more specific approach is required to determine the ubiquitination targets of the MtPHO2-E3 complex during SNF without P limitation.

## Data availability statement

The original contributions presented in the study are included in the article/**Supplementary Material**. Further inquiries can be directed to the corresponding authors.

## Author contributions

RH: conceptualization, methodology, investigation, formal analysis – tables and figures, writing - original draft, review and editing final manuscript; IT-J: methodology (RNA isolation and qPCR reactions); SJC: methodology (provided the biological material to isolate some of the mutant alleles) and editing; WS: supervision and editing; MU: review and editing final manuscript, supervision and funding. All authors contributed to the article and approved the submitted version.
