# Supplementary material for: Medicago truncatula PHO2 genes have distinct roles in phosphorus homeostasis and symbiotic nitrogen fixation
Source: Front Plant Sci. 2023 Jun 13;14:1211107. doi: 10.3389/fpls.2023.1211107 (PMC10319397; doi:10.3389/fpls.2023.1211107)
Supplement: Supplementary file 1 [file DataSheet_1.zip › Supplementary Figures.DOCX]

Supplementary Material

Medicago truncatula PHO2 genes have distinct roles in phosphorus homeostasis and symbiotic nitrogen fixation

^a^ Noble Research Institute LLC, Ardmore, Oklahoma, USA

* Correspondence: Michael Udvardi and Raul Huertas

Email: m.udvardi@uq.edu.au

Email: raul.huertas@hutton.ac.uk

The authors’ current address:

^1^ Environmental and Biochemical Sciences, The James Hutton Institute, Invergowrie, Dundee DD2 5DA, United Kingdom

^2^ Institute for Agricultural Biosciences, Oklahoma State University, Ardmore, OK, USA

^3^ United States Department of Agriculture, Plant Science Research Unit, St Paul, MN, USA

^4^ Department of Agronomy and Plant Genetics, University of Minnesota, St. Paul, MN, USA

^5^ Center for Plant Precision Genomics, University of Minnesota, St. Paul, MN, USA

^6^ Center for Genome Engineering, University of Minnesota, St. Paul, MN, USA

^7^ Queensland Alliance for Agriculture and Food Innovation, University of Queensland, Brisbane QLD 4072 Australia

# Supplementary Data

# Supplementary Figures and Tables

## Supplementary Figures


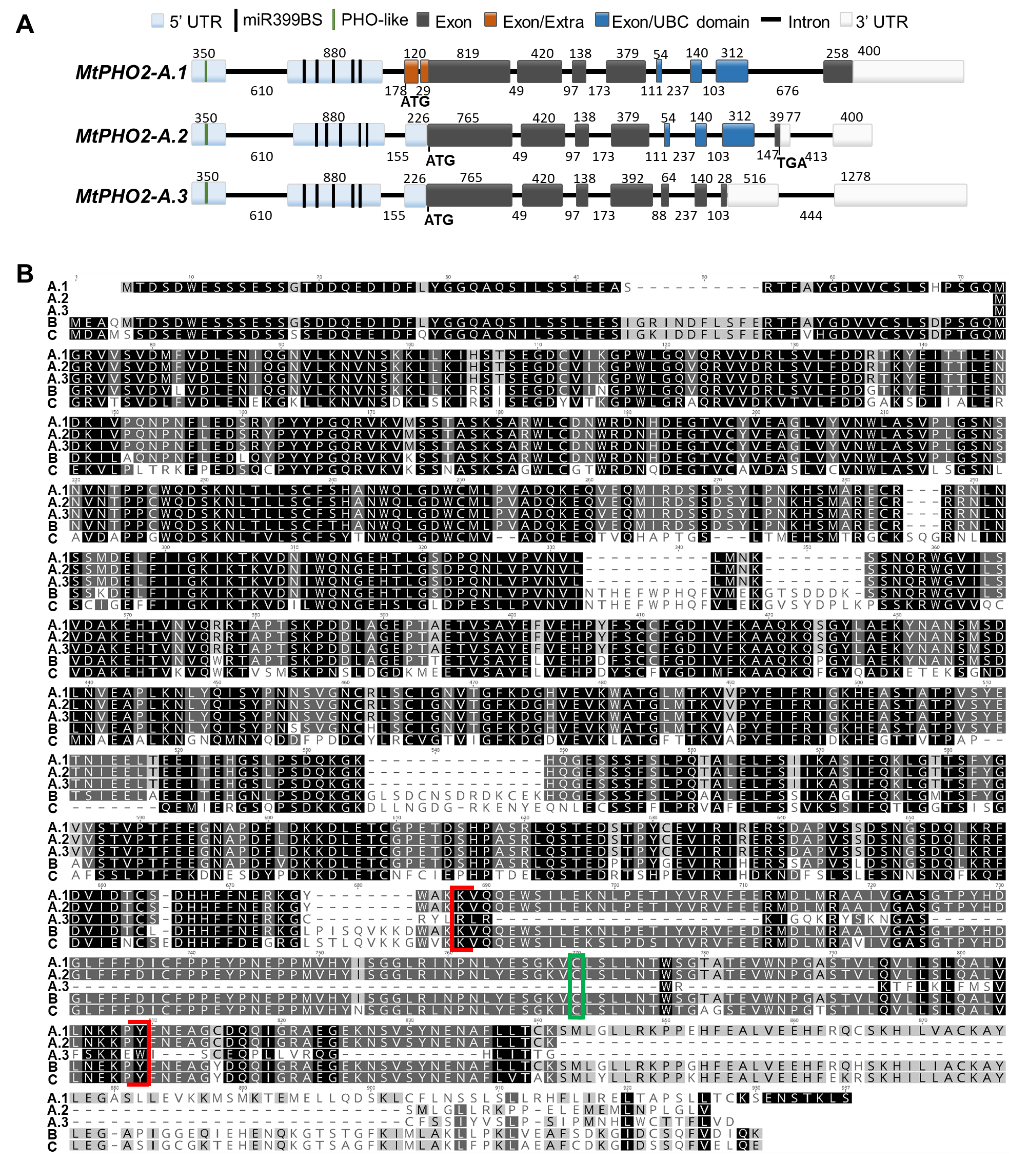


**Supplementary Figure 1.** **A)** Predicted gene structure of the three PHO2-A splicing variants present in the A17 Medicago truncatula genome (Mt4.0v1). Exons are shown as grey boxes or darker blue boxes, with the latter encoding the ubiquitin-conjugating (UBC) domain. Orange boxes indicate extra coding regions associated to the splicing events. UTRs are shaded light blue (5’ of the coding sequence) or white boxes (3’ of the coding sequence). The black and green lines in the UTR regions depict the position of the five potential miR399-binding sites (miR399BS) and PHO-like elements, respectively. Gene structures are drawn to scale, and the associated numbers indicate sizes (numbers above exons and below introns). **B)** Protein alignment of the five possible Medicago truncatula PHO2-like protein variants. Red square brackets indicate the E3 ligase interaction residues, and green square brackets indicate the E2 active site cysteine according to PROSITE database (https://prosite.expasy.org/). First 58 amino acids in PHO2-A1 correspond to the orange boxes in (A).

**
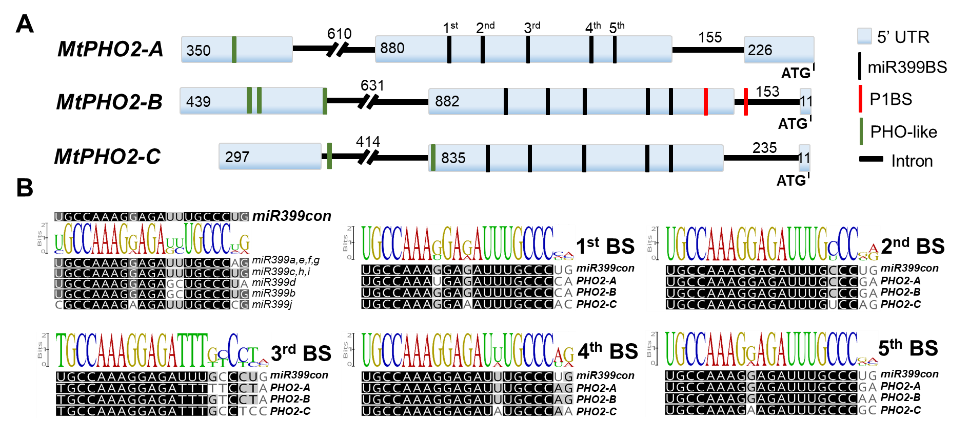
**

**Supplementary Figure 2.** **A)** Gene structure of the PHO2 5’ UTR regions present in the A17 Medicago truncatula genome (Mt4.0v1) and validated with RNA-seq results. Denoted are putative cis-regulatory motifs identified in the 5’ UTR regions: black lines show the position of the five potential miR399-binding sites (miR399BS), green lines depict the PHO-like element ([G(G/T/A) (C/T/A)GTGG], and red lines indicate possible PHR1 binding sites (P1BS)(GnATATnC). Structures are drawn to scale, and the associated numbers indicate sizes. **B)** Detailed view of the potential miR399BS in the 5′ UTR region. The consensus miR399 sequence (miR399con) was obtained from the 10 different genes (miR399a to j) and five miR399s variants obtained from miRBase ([www.mirbase.org](http://www.mirbase.org)). Different numbers indicate the order in the 5′ UTR regions according to A). Motif search and logo alignments were performed with Geneious Prime.

**
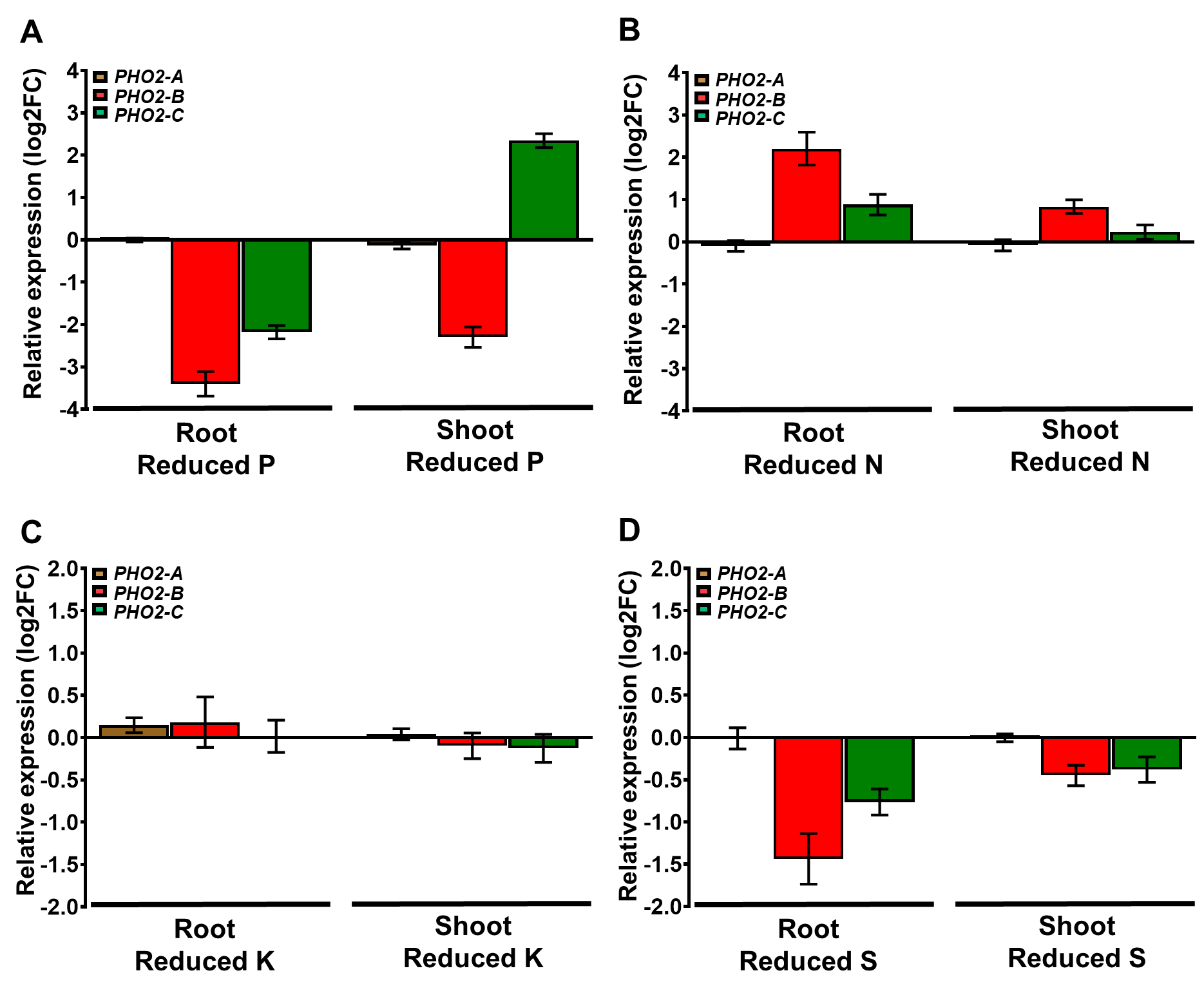
**

**Supplementary Figure 3.** Expression profiles of Medicago truncatula PHO2-like genes in roots and shoots of plants grown under different nutritional regimes. **A)** Phosphorus (P) limitation and **B)** Nitrogen (N) limitation. Data shown are the mean and SEM of three independent experiments. For each replicate, the log2-fold change (FC) was calculated using the FPKM values in the optimal condition for each nutrient as a reference. RNA-seq details are given in de Bang et al. (2017), Boschiero et al. (2020) and the M. truncatula SSP database (MtSSPdb) (<https://mtsspdb.zhaolab.org/database>).

**
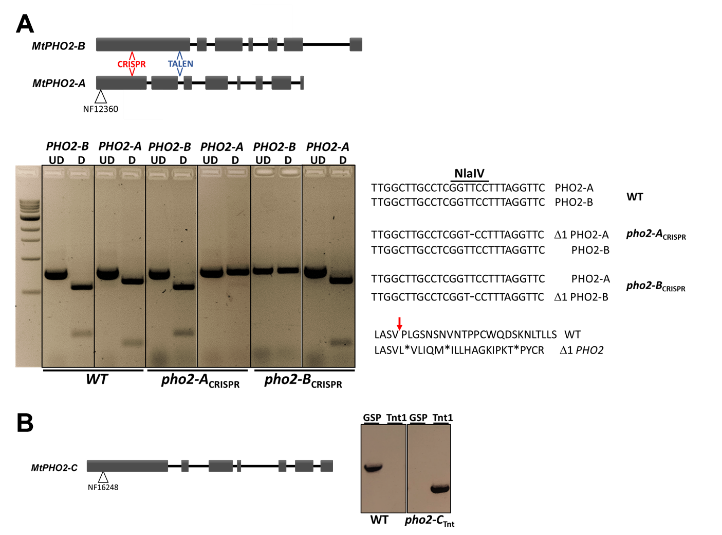
**

**Supplementary Figure 4.** Screening and identification of *Medicago truncatula* PHO2 mutant alleles. **A)** Top: maps of the two *MtPHO2* genes targeted for mutagenesis using CRISPR/Cas9 (red letters) and TALEN (blue letters). Bottom: the pho2-A_CRISPR_ and pho2-B_CRISPR_ mutants were identified by segregating plant WPT210-9 described in Curtin et al. (2017). Heritable transmission of the 1-bp mutations was confirmed by screening T1 plants by PCR-digestion assays (left) and sequencing (right). In both CRISPR mutants, the deletion in the coding region effectively disrupting the reading frame (indicated by the red arrow), generating the three stop codons (*) depicted in the amino acid sequence. UD means undigested PCR product, while D means digested PCR product, using the NlaIV restriction enzyme. The homozygous *Tnt1* line, NF12360 was used as a second mutant allele for PHO2-A (Curtin et al., 2017). The homozygous *pho2-B*_TALEN_ line (WPT52-4-8) described in Cermak et al. (2017) was used as a second mutant allele for PHO2-B. **B)** Seeds from the Tnt1 line NF16248 were screened by PCR using gene specific primers (GSP) to identify the homozygous mutant *pho2-C*_Tnt_ line at the *PHO2-C* locus. PCR primers used for screening are included in **Supplementary Table S2**.


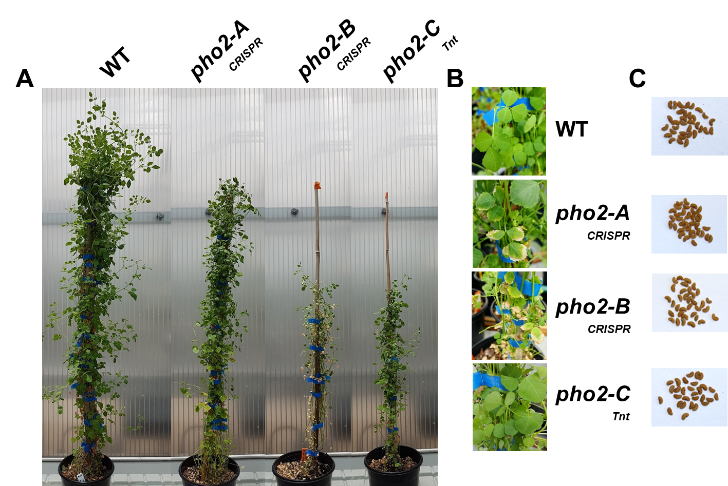


**Supplementary Figure 5.** Performance of the Mt*pho2* mutants under optimal nutritional conditions and grown in a greenhouse. **A)** Representative seven-week-old plants. **B)** Detailed view of old leaves with and without necrotic symptoms. **C)** Sample of seeds obtained from plants grown in a greenhouse.

**
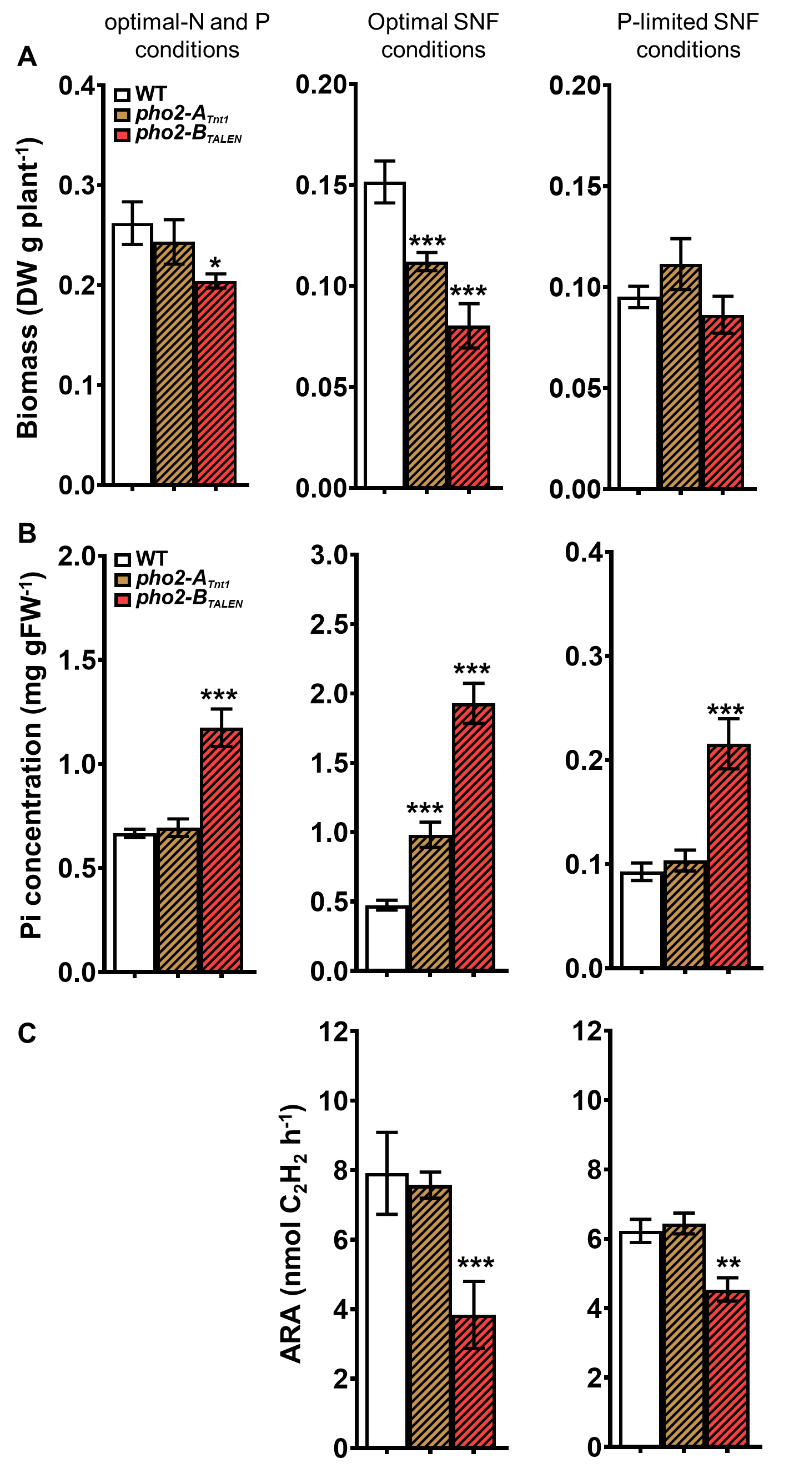
**

**Supplementary Figure 6.** Partial phenotypic characterization of *pho2-A*_Tnt1_ and *pho2-B*_TALEN_ mutant alleles. **A)** Plant dry weight. **B)** Free phosphate (Pi) concentration in old leaves. **C)** Acetylene reduction activity (ARA) of whole nodulated roots under optimal-N and P conditions (left column plots), optimal SNF conditions (middle column plots), and P-limited SNF conditions (right column plots). Data shown are the mean and SEM of two independent experiments (n=3 / experiment). Asterisks indicate significant differences between the wild type and the mutants calculated using two-tailed Student’s t-tests (*p < 0.05, **p < 0.01, ***p < 0.001).

## Supplementary Tables

**Supplementary Table 1.** List of PHO2 proteins identified in the databases.

**Supplementary Table 2.** List of primers used for genotyping and quantitative PCR (qPCR) analysis.

Boschiero, C., Dai, X., Lundquist, P. K., Roy, S., Christian de Bang, T., Zhang, S., . . . Zhao, P. X. (2020). MtSSPdb: The Medicago truncatula Small Secreted Peptide Database. *Plant Physiology, 183*(1), 399-413. <https://doi.org/10.1104/pp.19.01088>.

Cermak, T., Curtin, S. J., Gil-Humanes, J., Cegan, R., Kono, T. J. Y., Konecna, E., . . . Voytas, D. F. (2017). A Multipurpose Toolkit to Enable Advanced Genome Engineering in Plants. *Plant Cell, 29*(6), 1196-1217. <https://doi.org/10.1105/tpc.16.00922>.

Curtin, S. J., Tiffin, P., Guhlin, J., Trujillo, D. I., Burghart, L. T., Atkins, P., . . . Young, N. D. (2017). Validating Genome-Wide Association Candidates Controlling Quantitative Variation in Nodulation. *Plant Physiology, 173*(2), 921-931. <https://doi.org/10.1104/pp.16.01923>.

de Bang, T. C., Lundquist, P. K., Dai, X., Boschiero, C., Zhuang, Z., Pant, P., . . . Scheible, W. R. (2017). Genome-Wide Identification of Medicago Peptides Involved in Macronutrient Responses and Nodulation. *Plant Physiology, 175*(4), 1669-1689. <https://doi.org/10.1104/pp.17.01096>.
